# The ECM Moves during Primitive Streak Formation—Computation of ECM Versus Cellular Motion

**DOI:** 10.1371/journal.pbio.0060247

**Published:** 2008-10-14

**Authors:** Evan A Zamir, Brenda J Rongish, Charles D Little

**Affiliations:** Department of Anatomy and Cell Biology, School of Medicine, The University of Kansas Medical Center, Kansas City, Kansas, United States of America; Duke University Medical Center, United States of America

## Abstract

Galileo described the concept of motion relativity—motion with respect to a reference frame—in 1632. He noted that a person below deck would be unable to discern whether the boat was moving. Embryologists, while recognizing that embryonic tissues undergo large-scale deformations, have failed to account for relative motion when analyzing cell motility data. A century of scientific articles has advanced the concept that embryonic cells move (“migrate”) in an autonomous fashion such that, as time progresses, the cells and their progeny assemble an embryo. In sharp contrast, the motion of the surrounding extracellular matrix scaffold has been largely ignored/overlooked. We developed computational/optical methods that measure the extent embryonic cells move relative to the extracellular matrix. Our time-lapse data show that epiblastic cells largely move in concert with a sub-epiblastic extracellular matrix during stages 2 and 3 in primitive streak quail embryos. In other words, there is little cellular motion relative to the extracellular matrix scaffold—both components move together as a tissue. The extracellular matrix displacements exhibit bilateral vortical motion, convergence to the midline, and extension along the presumptive vertebral axis—all patterns previously attributed solely to cellular “migration.” Our time-resolved data pose new challenges for understanding how extracellular chemical (morphogen) gradients, widely hypothesized to guide cellular trajectories at early gastrulation stages, are maintained in this dynamic extracellular environment. We conclude that models describing primitive streak cellular guidance mechanisms must be able to account for sub-epiblastic extracellular matrix displacements.

## Author Summary

Swirling dance-like patterns of cellular motion accompany the formation of a vital embryonic structure in birds and mammals called the primitive streak, which is located where the future vertebral column will form. The primitive streak is considered the “organizing center” of the embryo, because during early embryonic life the cells in the top-most tissue layer move through the streak and generate the middle and bottom-most embryonic tissue layers, in a process called gastrulation. Despite the extreme importance of gastrulation, the mechanics driving the formation of the primitive streak are not well understood. Here, we use time-lapse microscopy in living embryos to study cellular motion, as well as the motion of the connective tissue fibers, beneath the cells. We show for the first time that the nonliving connective tissue fibers do not form a static scaffold over which cells move—indeed, the fibers are engaged in the same “dance” as the upper layer of cells. Our “composite motion” movies (cell + fibers) advance understanding of how embryos organize their shape during a critical period that a famous developmental biologist, Louis Wolpert, called “The most important day of your life.”

## Abstract 3

Detailed in vivo video microscopy of the motion of cells and connective tissue in the primitive streak of avian embryos undergoing gastrulation reveals that the connective tissue moves in concert with the cells.

## Introduction

Well-recognized bilateral, countercurrent, vortical patterns of epiblast cellular movements accompany formation of the amniote primitive streak (PS)—the organizing center for gastrulation [1–3]. Two recent time-lapse microscopy studies of chicken PS formation have provided the first descriptions of these movements at cellular resolution by using electroporation to transfect and label individual epiblastic cells with green fluorescent protein (GFP) (or equivalent) plasmids [4,5]. Although the movements described in classical and modern studies appear similar [1,2,4], the biophysical mechanisms driving these movements are unknown. Indeed, even the proposed models conflict. Whereas Voiculescu [4] and co-workers hypothesize that epithelial cell intercalation drives PS formation, Chuai et al. [5] disagree that intercalation is the primary mechanism and instead suggest that chemotaxis acting through fibroblast growth factor signaling pathways may be responsible.

In striking contrast to extensive studies on cellular motion—there are no published time-lapse data regarding extracellular matrix (ECM) dynamics during PS formation. Electron microscopy and immunofluorescence studies demonstrated decades ago the presence of a nascent basement membrane-like structure, which we refer to as the sub-epiblastic ECM (SE ECM), containing, at least, fibronectin (FN) [6,7] and collagen [7]. This SE ECM (see Results for an operational definition of the term) is present as early as a freshly laid egg [7,8]. Using ultrastructural markers, including FN antibodies conjugated to peroxidase, Sanders [9] found that the SE ECM is transported medially to the PS with the epiblastic cells. Almost two decades later, however, Bortier and colleagues [10] revisited this issue and came to a different conclusion. They examined radiolabeled embryonic quail cells grafted into the epiblasts of chicken blastoderms and asserted that whole groups of epiblastic cells slide across (move relative to) the SE ECM, thus contradicting Sanders' earlier findings.

Our time-lapse experiments show that immunofluorescent antibodies can be used to track dynamic movements of ECM in vivo [11–14]. More recently, we demonstrated that, by analyzing mesodermal cell and ECM movements simultaneously, cell-autonomous (CA) movements (i.e., “true” cell motility or active migration) can be directly separated from bulk (tissue-level) morphogenetic movements that convect cells passively [11]. Here, we use time-lapse techniques to study the relative motion of individual epiblastic cells and SE ECM in living avian embryos during early PS stages. Our data definitively show that the SE ECM exhibits an organized and extensive pattern of motion, including vortex-like movements, convergence to the midline, and extension along the embryonic anterior-posterior axis—thus supporting Sanders' observations [9], not those of Bortier et al. [10]. For the most part, the epiblastic sheet moves in concert with SE ECM. Furthermore, our data suggest that experimental or quantitative models for cellular guidance mechanisms—whether they are based on cell–cell intercalation or chemotaxis—must be able to account for the persistent motion of the SE ECM we document below.

## Results

### Operational Definition of SE ECM

SE ECM is operationally defined here as a moving meshwork of fibronectin FN fibrils, which lie subjacent to the epiblastic epithelium. In formal usage the term basement membrane denotes a specific kind of an organized ECM, associated with the basal surface of a mature epithelium, which typically contains laminins, specific collagens, and proteoglycans. To avoid the implication that the immunofluorescent FN monitored in our study is a proven constituent of a bona fide basement membrane, we use a more neutral term, sub-epiblastic extracellular matrix or SE ECM.

### FN Immunofluorescent Localization in the SE ECM and PS

**Figure 1:**
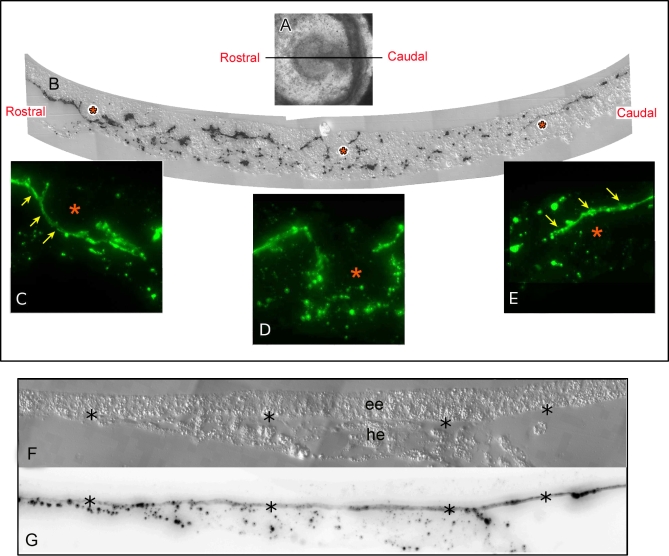
The Distribution of Immunoreactive Fibronectin in a HH Stage 2 Quail Embryo (A and B) A whole-mounted embryo, en face, with a line indicating the cranial–rostral orientation of a section that passes through the PS (A). One such semi-thin section (10 μm) in which the differential interference contrast image is superimposed on the corresponding immunofluorescent fibronectin pattern (B). (C–E) Higher-resolution (40×) immunofluorescent images. Asterisks denote equivalent positions in (B). Note that the tissue at the extreme cranial and caudal ends of the PS contains semi-continuous bands of FN immunofluorescence (arrows; C and E); in contrast, the middle region of the PS (*, D) exhibits an irregular pattern, with bright clumps of FN distributed throughout the thickness of the section. (F and G) Two views of a semi-thin serial section from the same embryo. This parallel section was taken from a more lateral position, compared to the sister section depicted in (B) (i.e., lateral to the PS). Note that tissue taken from this more lateral position displays a uniform, continuous band of FN immunofluorescence (asterisks, G), which is immediately subjacent to the epiblastic epithelium (ee) (F). The cell layer situated inferior to the asterisks, is the hypoblastic epithelium (he). Comparing the midline section (B) to the lateral section (G) demonstrates a distinct difference in the respective FN immunostaining profiles. The black asterisks (F and G) are in equivalent anatomical positions.

To verify that microinjection of FN-monoclonal antibody (mAb) into live embryos results in SE ECM labeling (as opposed to diffuse staining or cellular uptake), and to characterize the localization pattern of FN immunofluorescence, several in vivo–labeled embryos, Hamburger-Hamilton (HH) stage 2 or 3 (PS-stages) were fixed and sectioned longitudinally (Figure 1). A section through the cranial–caudal midline of one embryo, at approximately HH3, is shown (Figure 1B). As expected, the semi-thin (10 μm) section reveals robust fibronectin immunoreactivity (Figure 1B–1E). The central region of the section that passed through the PS exhibits discontinuous clumps of FN throughout its thickness when viewed at higher magnification (*, Figure 1D).

In contrast, when a sister section from a lateral position is examined, the FN is present as a continuous cranial–caudal band (Figure 1G). These two distinct in vivo labeling patterns show that the FN-containing ECM in the “middle” of the primitive streak region is less uniform compared to the “basement membrane”-like structure present in more lateral regions (also see [6,15]). The medial-to-lateral staining differences are consistent with primitive streak tissue engaging in relatively greater biological motion compared to tissue on each side of the streak.

### SE ECM Exhibits Dynamic Movement Patterns

**Figure 2:**
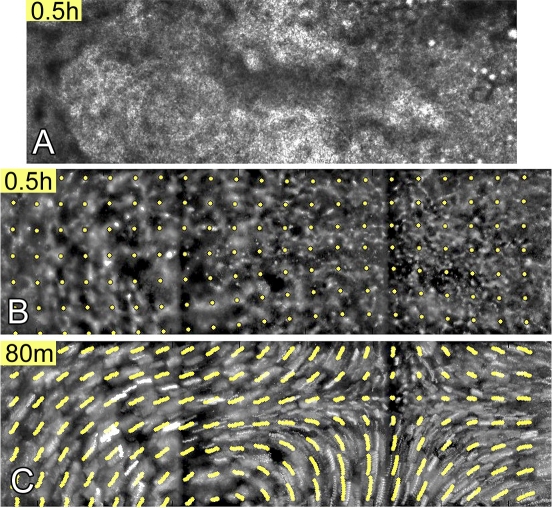
A Time-Dependent Visualization of Fibronectin-Derived SE ECM Movements Starting at Approximately HH Stage 2 (A) En-face, brightfield, view of an embryo near the beginning of the time-lapse sequence (30 min). (B) The corresponding immunofluorescent fibronectin image seeded with a regular array of virtual material particles. (C) Approximately 80 min of the subsequent time-lapse recording. The yellow trajectories represent the motion of virtual material particles over ten time-points, as determined by the PIV analysis. See Video S1 for an 8-h time-lapse sequence of the fibronectin-derived virtual material particle plots superimposed upon the motion of immunofluorescent fibronectin fibers, and see Video S2 for the brightfield time-lapse sequence of this embryo.

To visualize SE ECM motion, we performed particle image velocimetry (PIV) analysis on a set of FN-immunofluorescent image frames from a time-lapse experiment (Video S1; see Methods and Materials). The underlying assumption is that FN is an integral SE ECM component and therefore can be used as a passive fiduciary marker; further, FN fibrils are not self-propelled objects. Using the PIV-determined incremental displacement field, we “seeded” the first time-point image (also called material or reference frame) with an array of evenly spaced “virtual material particles” (VMP) shown in Figure 2B, and then calculated the spatial (eulerian) trajectories for each set of particle coordinates (Figure 2C).

**Figure 3:**
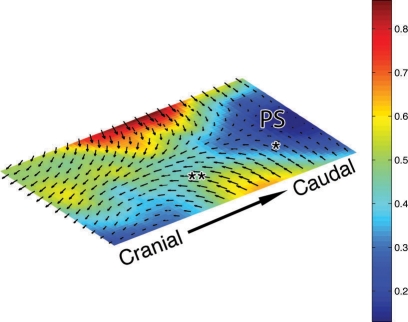
A Graphical Depiction of Instantaneous Fibronectin Velocity (Time-Averaged over the Course of 2 h) Showing the Normalized Direction Vectors (Arrows) and Speed (Indicated by Color Contours) of FN Motion during PS Formation The velocity field is (mostly) symmetric about the longitudinal axis of the PS (i.e., bilateral symmetry). The units for the scale bar are speed (in μm/min). High-resolution movies of cells and FN in the regions denoted by asterisks are included as Videos S5 (*) and S6 (**).

Given the spatial and temporal heterogeneities that exist between different specimens, as well as non-uniformity of FN labeling, it would be difficult, and perhaps less informative overall, to provide an average or “ensemble” map of FN movement. Instead, for the purposes of the present study, and similar to the approach taken by Voiculescu et al. [4], we provide representative results for a single uniformly labeled HH2 embryo (Figure 2). The specimen demonstrates the general pattern of FN movement (Figure 2C), which was consistently observed over the course of several recordings (*n* > 10; see Video S2 for the corresponding brightfield time-lapse sequence). Figure 3 shows a time-averaged calculation for the instantaneous FN velocity field surrounding the PS.

**Figure 4:**
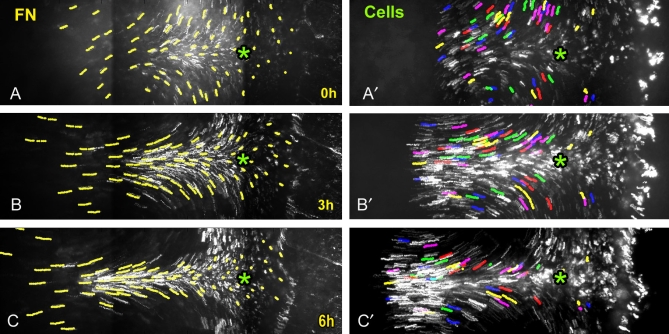
A Comparison of Fibronectin and Epiblastic Cellular Movements during PS Formation of a HH2 Quail Embryo The left-hand images (A–C) depict two datasets representing FN motion. Each image shows a “running projection” of ten consecutive time-lapse frames (approximately 80 min) from a recording of the immunofluorescent FN channel (white). Overlaid upon the (background) images are the corresponding FN-virtual material particle trajectories (yellow) that were computed using PIV analysis. The running projections are shown beginning at 0 h (A/A′), 3 h (B,B′), and 6 h (C/C′). The two datasets represent distinct means of computing the motion of a sub-epiblastic extracellular matrix constituent. The right-hand images (A′–C′) also depict two datasets. Here, each image shows a running projection of ten time-lapse frames from a recording of H2B-GFP labeled epiblastic cell nuclei (white) at three different time-points corresponding to those in the left column (0, 3, 6 h). These images are overlaid with approximately 46 individual cell trajectories (colored lines) that were acquired by manual tracking using an Image-J plugin. The green asterisks depict the same anatomical position in the embryo at each respective time point (A/A′, B/B′, C/C′). The motion pattern is similar among all four methods of analysis—compare the right and left images at each time point.

The main results in Figures 2 and 3 are the following: (1) The displacements of VMP lateral to the PS are large (hundreds of micrometers) and generally directed toward the midline of the embryo. These movements clearly represent “convergence” of the SE ECM. (2) VMP cranial and caudal to the PS move in anterior and posterior directions, respectively, coinciding with “extension” movements of the embryo, and the PS itself. (3) Bilateral, countercurrent, vortical movements are clearly visible both in the VMP trajectories and in time-projected FN immunofluorescence motion—the vortex-like motion is especially apparent during the early stages of PS formation (Figures 2C and 4). It should be noted that VMP trajectories are a concise, unbiased means of visualizing and describing SE ECM motion. The time-lapse data (Video S2) show that equivalence between VMP and FN displacement patterns is easily discerned.

### The Epiblast Moves in Concert with SE ECM

To examine the relative movements of epiblast cells with respect to the SE ECM, some embryos were electroporated with GFP and subsequently immunolabeled with FN-mAb by microinjection (see Methods and Materials; Video S3). As in the previous section the results for a single representative embryo are shown (Figure 4). The data in Figure 4A–4C allow an observer to compare the FN-derived virtual material particle(s) (FN-VMP) trajectory patterns to the actual FN displacement fields at three time points. The corresponding Figure 4A′–4C′ show the total GFP projected cellular trajectories (white) plus individually tracked epiblastic cells (colored lines). Comparisons can be made at any arbitrary region of the embryo and at multiple time points. The results clearly indicate that, for both individual cells and FN-VMP, the trajectories are approximately equivalent, based on direction and path length (i.e., total cumulative distance traveled)— strongly suggesting that the cells and their nearby ECM move as a tissue composite. In other words, the time-lapse data show that the motion of the SE ECM is very similar to the epiblastic cellular motion pattern. Furthermore, the degree of epiblastic CA motility near the PS is limited at these stages, with most of the motion confined to tissue convection. These statements are true whether one compares cellular motion to the FN-VMP displacements (Figure 4A–4C) or to the actual excursions of the fluorescent FN fibrils (Video S4).

**Figure 5:**
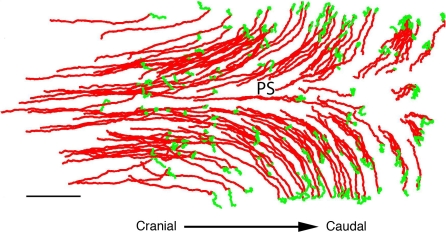
Total (Red) and CA (Green) Epiblastic Cellular Trajectories (*n* = 173 cells) for the Same Embryo Analyzed in Figure 4 Scale bar = 100 μm. As explained in the text, the CA trajectories were calculated by subtracting the contribution of FN velocity (e.g., convection) at each cell coordinate as a function of time during the experiment.

Similar to our previous analyses [11], we define CA velocity as the difference or residual between the total (spatial) cell velocity and PIV-calculated (FN) velocity field at each cell coordinate (location) as a function of time. The total and CA trajectories are then determined simply by summing the (incremental) velocity vectors for each cell during the course of a time-lapse movie (see, for example, Video S4). Such an analysis immediately reveals that the CA trajectories constitute only a small fraction of the total epiblastic cell displacements (Figure 5). In other words, most of the epiblastic displacement can be attributed to the FN motion.

**Figure 6:**
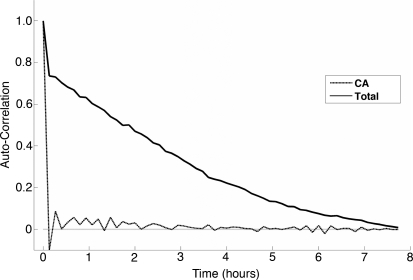
Auto-correlation Scores Were Calculated for the Total and CA Velocity Vectors for Each Tracked Cell The auto-correlation data were averaged over all the cells that were tracked. Here, the relatively slow decay of the total cellular velocity indicates the highly persistent (oriented) contribution of the FN velocity component, as opposed to the (almost) instantaneous decay toward zero correlation for the CA velocity, which strongly indicates the lack of persistence of CA motility. In other words, after the convective FN velocity is subtracted from the total cellular displacements, the residual component of cell directionality appears to be essentially random.

In a previous study, we found that the CA speed for gastrulating mesodermal cells was in the range 0.8–1.3 μm/min [11], which is significantly higher than our calculation for epiblastic CA speed (0.2–0.3 μm/min; *n* = 173 different cells tracked over a total of 7,621 time points or frames). Furthermore, autocorrelation analysis of individual cell trajectories revealed that the CA component of epiblastic cell velocity exhibits very weak correlation compared to the total cellular velocity (Figure 6). These results suggest that (1) CA motility is not highly aligned in any particular direction with respect to the ECM fiber motion over any significant time period, and (2) the cells undergo a random walk with no apparent directional bias or persistence. To confirm these findings, we investigated the CA speed and autocorrelation statistics for groups of closely “neighboring” cells in several different regions of the epiblast. We were unable to find any consistent differences between individual cells within each group or among different groups (unpublished data). Moreover, two movies show cells and FN at high resolution within two distinct anatomical regions: (1) the caudo-lateral portion of the PS, which exhibits relatively small tissue displacements (Video S5; corresponding to * in Figure 3); and (2) the mid-lateral PS where there is extensive convergence of the tissue toward the midline of the embryo (Video S6; corresponding to ** in Figure 3). These time-lapse data strongly reinforce our suggestion that total cellular motion is directly correlated to tissue motion (FN), without regard to whether the anatomical region is undergoing small or large macroscopic movements.

## Discussion

Convergence and extension movements are well studied in several vertebrates, including *Xenopus* [16–20], zebrafish [21–24], and more recently chicken [4,5,25]. Our “real-time” in vivo motion analysis of fibronectin displacements adds important new concepts to understanding biological motion patterns in the early embryos of warm-blooded animals, namely: (1) In primitive streak stage avian embryos, extracellular matrix movements are substantial, i.e., motion occurs across tissue-level length scales; (2) extracellular matrix fibrils exhibit both vortical and “convergent extension” motion patterns, heretofore attributed solely to epiblast or mesodermal cellular layers [17,21]; and (3) epiblastic cells move in close concert with the subjacent extracellular matrix. These results have profound implications for our understanding of primitive streak formation and gastrulation, and they will influence how future investigators study embryonic morphogenetic movements and tissue patterning. In particular, the relative contributions of cell-autonomous motility versus composite tissue motion (cells + matrix) will have to be explained.

There is a large literature regarding how chemical gradients of protein morphogens, such as fibroblast growth factor and Wnt family members, generate molecular signals that drive cellular motion during early embryogenesis [1,5,23,26,27]. Such chemical gradient molecules are presumed to function in the extracellular space, i.e., in a compartment external to the target cells. Nevertheless, changes in embryonic morphology are virtually always attributed (solely) to changes in cellular “migration” patterns. In contrast, our data, which demonstrate a strong correlation between cellular and extracellular matrix movements, raise interesting questions regarding how putative chemical morphogen gradients are created/maintained at early stages. Our data raise the obvious question: do “dynamic” chemical gradients look different from the static gradients depicted in virtually all diagrams?

It is reasonable to assume that, for any morphogen gradient to operate, embryonic cells must be exposed at the appropriate time and anatomical position [28]. Thus, a critical design component of any gradient-based mechanism is whether cells move relative to, or with, the ECM. Our present and recent data show that both the epiblast and mesoderm engage in extensive tissue-level movements involving both cells and closely associated ECM [11,13,14] — thus implying the movement of the gradients themselves. To our knowledge, there are no empirical studies or mathematical conjectures addressing how chemical morphogen gradients are created and/or maintained in such a dynamic tissue/ECM milieu. For example, Painter et al. [29] developed a mathematical model for primitive streak formation driven by chemotaxis. The authors use reaction-diffusion equations to show that if cells in the PS region produce a gradient in a certain way, they can produce elongation and regression of the streak. Although mention of an “underlying domain” is made, there is no mention of the possibility that the domain is moving. Oster and his colleagues recognized this problem almost three decades ago: “Moreover, each cell must be able to ‘read' the local concentrations of morphogens accurately and differentiate accordingly. Thus the cell pattern is but a passive reflection of a pre-existing morphogen pattern. In effect, the problem of cell patterns has been replaced by the physical-chemical problem of distributing the morphogens appropriately by intercellular diffusion and reaction” [28].

Based on experimental results, Chuai and co-workers [5] have recently proposed that cells are “attracted” to or “repelled” from the midline, according to their position along the morphogen gradient field. The implicit assumption of this hypothesis is that the ECM is used as a substrate for cell migration. If Chuai et al. are correct, as the cells progress toward the streak, cell traction forces would tend to push matrix fibers away from the midline (action–reaction). In contrast, our results show that epiblastic cells either are adherent to the SE ECM or remain closely affiliated with a particular ECM assembly (see Videos S5 and S6). At the very least, our data suggest that somehow epiblastic cells pull the matrix toward the midline, rather than crawl across it. Thus, the empirical data cast doubt on a simple chemoattractant hypothesis, because the cells do not appear to demonstrate any particular directional bias that is independent of the large-scale tissue movements represented by SE ECM displacements.

What, then, causes the movement of SE ECM? Voiculescu et al. hypothesize that the (cellular) epiblast movements are a result of midline intercalation of cells, explaining that the “additive effects of these small local displacements cause distant cells to move faster” [4]. It is important to note that the Voiculescu model differs from the “standard convergent extension” model of Keller and colleagues (see [20] for recent review) in two significant ways: (1) Voiculescu et al. propose that convergent extension/intercalation begins much earlier in amniotes (during primitive streak formation) than non-amniotes, such as *Xenopus*, and that this early intercalation mechanism may be unique to amniotes; and (2) according to Keller [20], convergent extension in *Xenopus* is thought to be due to forces generated by the deep, non-epithelial mesenchymal cells in both the mesoderm and neural regions, not the overlying epithelia/epiblast—thus, Keller and Voiculescu et al. differ on the time and place (tissue layer) convergent extension affects morphogenesis in bird versus frog embryos. The two models are not mutually exclusive, and the discrepancies may be due to real differences between amniotes and non-amniotes.

The forces produced by the intercalation of midline cells must be large enough to “drag” the SE ECM, along with the epiblastic cells, toward the primitive streak; unfortunately, the magnitude and direction of cellular intercalation forces in embryos is unknown at this time [20]. Furthermore, it is not known how the tensile forces are distributed between integrin-mediated cell–matrix adhesions and cadherin-mediated cell–cell adhesions [20].

Brodland has recently created a computational model (finite element) for convergent extension, which includes an ECM substratum [30]. This model illustrates how tissue flow fields, similar to those we and others have observed [2,4,5], can hypothetically be produced by the concerted effort of active lamellipodia-derived forces driving mediolateral intercalation of cells. Brodland's concept fits with the view of Keller and colleagues, who hypothesize that the ECM substrate should be relatively flexible and deformable to allow convergent extension [20]. Our dynamic imaging data clearly demonstrate that the SE ECM undergoes striking deformations during PS formation, concurrent with extensive assembly and coalescing of a fibrous fibronectin architecture. Thus, our data are consistent with the recent model of convergent extension driven by mediolateral cell intercalation, as proposed by Voiculescu et al. [4].

Both the above-mentioned mediolateral intercalation model and the chemotaxis model propose that epiblastic movements are due primarily to locally active cell behaviors, while the matrix plays a purely passive role. We favor a different hypothesis regarding collective cellular + ECM motion, and stress that other, more global forces may be operating. Our new data are consistent with the following possible mechanisms for the epiblastic movements described in this study: First, concerted cellular and ECM movements may result from large-scale tissue movements that act to converge and extend the entire embryo, even though the forces for these movements are not generated in the particular regions of the epiblast that were analyzed in this study. To give one example, convergent extension acting in the anterior neural plate region, combined with anchoring of the caudal portion of the streak near Koller's sickle, could result in extension of the entire embryo and thus cause the concerted epiblast/ECM movements that were observed. This situation would be analogous to stretching a thin strip of rubber by applying forces at the two ends. Second, a potential force-generating mechanism for driving large-scale tissue movements involves the biochemical reactions of ECM fiber assembly. At the present time, there is little evidence that matrix fiber assembly is capable of deforming embryonic tissue—on the other hand there are very few, if any, studies that address this hypothesis. Our preliminary data regarding other extracellular matrix constituents (laminin, collagen IV, and fibrillin 2) indicate primitive streak stage motion patterns similar to those of fibronectin fibrils described above (unpublished observations). Thus, the physical–chemical and biochemical reactions that drive fiber assembly of multiple extracellular matrix constituents might collectively exert compression or stretching forces on soft embryonic tissues.

The present data show that we have established a method to quantify autonomous cellular displacement versus passive tissue convection. The next steps are to (1) identify biomechanical forces acting at both cellular and tissue-level length scales, including both adhesive and pushing/pulling forces; (2) define the material (engineering) properties of the epiblastic tissue (cells and ECM); and (3) determine the universality of ECM fibril motion during primitive streak formation (i.e., Do all matrix components share similar motion patterns with FN?). Only then will we be in a position to understand the physics underlying propulsion of the ECM scaffold. This formidable multi-scale problem will be a fruitful area of investigation for bioengineers, who can devise means to measure forces and mechanical properties in vivo; and also for biomathematicians and biophysicists who construct models and computer simulations of embryonic morphogenesis. It is now clear that understanding ECM motion will be required to answer the rhetorical question posed by John Trinkaus: What are “the forces that shape the embryo” [31]?

## Materials and Methods

### Avian embryo preparation.

Locally raised fertilized quail eggs (*Coturnix coturnix japonica*, Ozark Eggs, Stover, Missouri) were incubated for approximately 4–5 h at 38 °C. The embryo was dissected from the egg, mounted on filter paper rings, placed ventral side up on a semi-solid mixture of agar/albumen (egg whites) (modified after the method of New [32–34]), and cultured at 38 °C until approximately HH stage 2 [35] or the beginning of PS formation. During a time-lapse experiment, embryos were incubated in a custom-built, temperature- and humidity-controlled chamber mounted on a microscope stage [13,36,37].

### Ex ovo transfection of DNA plasmids.

H2B-GFP nuclear-targeted plasmid DNA constructs (courtesy of Dr. R. Lansford, Caltech, Pasadena, California) were expressed in bacterial culture and then purified using the Endo-Free Plasmid Purification Maxi Prep (QIAGEN). Full details of the ex ovo electroporation method for pre-gastrulation stage avian embryos have been recently published [38], and are similar to the methods used in Voiculescu et al. [4] and Chuai et al. [5]. GFP cell labeling is typically visible within 2–3 h after electroporation.

### Immunofluorescent labeling of fibrillar ECM.

The mAb to avian FN, B3D6 (Developmental Studies Hybridoma Bank, University of Iowa), was directly conjugated with Alexa-555 fluorochrome (Molecular Probes). GFP-transfected embryos were taken out of the incubator after 2–3 h, and several bolus microinjections of the fluorescent mAb were made carefully around the edge of the PS approximately 1 h before the start of a time-lapse experiment to allow for diffusion and binding to antigen [11,13,39].

### Digital wide-field time-lapse microscopy.

The details of our digital time-lapse microscopy system have been fully described [36,37]. Briefly, a computer-controlled wide-field (10× objective) epifluorescent microscope equipped with motorized stage and cooled CCD digital camera was used to acquire 12-bit grayscale intensity images at multiple *x-y* locations (tiles) and focal planes (*z*-stacks). Both brightfield and fluorescent images were acquired using separate fluorochrome filters. Images were acquired at approximately 8 to12 frames per minute (fpm), depending on the number of tiles and illumination modes. Following an experiment, image processing, including focal plane collapsing (*z*-projection) and tile mosaicking (“stitching”) were performed to create high-resolution whole-embryo 2-D time-lapse sequences for subsequent use in cell tracking and PIV analysis.

### PIV analysis of SE ECM motion.

Complete details of the implementation and validation of the PIV method in early avian embryos have been reported elsewhere [11,13]. Briefly, the commercially available mathematical programming language Matlab (Mathworks, Natick, Massachusetts) is used to implement the PIV algorithm. Given a pair of sequential images from a FN time-lapse image sequence, we desire a maximum-likelihood estimate for the incremental displacement field. To do this, we use a two-step linear predictor-corrector approach [40,41]. First, a coarse or predictor displacement field is found using normalized cross-correlation operating on equally spaced overlapping square blocks of pixels (sub-windows). In the subsequent corrector step, the coarse displacement predictions are used to offset the sub-windows by an integer pixel amount, and the cross-correlation procedure repeated. In this iteration, however, the sub-window size is reduced by a factor of two, and the search radius reduced to a size on the order of the error made by the initial predictor approximation, typically 4 pixels. Finally, a thin-plate spline approximation is used to obtain a smoothly varying and continuous displacement field, which can then be numerically evaluated at any arbitrary location in each time-lapse image frame.

Using the PIV-determined incremental displacement field, we then “seed” the first image with an array of evenly spaced “virtual material particles” (VMP) and calculate the spatial (eulerian) trajectories for each set of particle coordinates. To do this, we evaluate and store the value for the incremental displacement field at the (current) position for each VMP, then perform an “update” whereby the particle coordinates are moved by that amount of displacement for each image (time-point) in the sequence. The result is that the movement of each VMP is coordinated precisely with the average local FN motion, but not necessarily one particular feature (e.g., single fibril). We then graphically overlay the VMP onto the original images and observe their motion with respect to the embryo, as if the VMP were leaves flowing in a stream. Performing a “running projection” of several images at each time-point creates trajectories that are readily visualized. The advantages of using the PIV approach are that the selection of VMP coordinates and calculation of particle trajectories are automated and highly precise, which eliminates human bias and error that can occur with manual particle tracking, especially for fibrillar entities, such as FN [12,13]. Note that the term “virtual” simply denotes that the particles are calculated entities, as opposed to the (real) cell trajectories discussed in the next section, which represent the movement of particular cells.

### Epiblastic cell tracking.

Time-lapse image sequences for GFP-labeled embryos were loaded into the freely available ImageJ (http://rsb.info.nih.gov/ij/) program, and epiblastic cell tracking and cell trajectory visualization was performed using the “Manual Tracking” plugin (http://rsb.info.nih.gov/ij/plugins/track/track.html). Cells were tracked either from the start of the time-lapse sequence or once GFP expression became clearly visible. Similarly, cells were tracked for as long as they were clearly identifiable and could be distinguished from other cells.

### Velocity auto-correlation.

In general, the auto-correlation function (ACF) measures the temporal persistence (or randomness) of a signal by correlating data points at one chosen time with every other time point and then averaging over all times. To quantify the persistence of cellular velocities in the epiblast (either with or without ECM motion subtracted), we computed the velocity ACF for each cell, C(τ) = 〈 **v** (t) • **v** (t + τ) 〉, where τ is called the “delay time,” **v** is the velocity vector, and the symbols 〈 〉 indicate averaging over all times t. C was unity-normalized by the maximum value of the ACF, which always occurs at τ = 0.

## Supporting Information

Video S1 Movement of Immunofluorescent Fibronectin and Computationally Determined Virtual Material Particles during Early PS Formation This time-lapse movie shows the movement of immunofluorescent fibronectin and computationally determined virtual material particles during convergent/extension movements of early primitive streak formation. Figure 3 shows the vortex-like FN movements at the beginning of the movie.
(9.53 MB MOV)

Video S2 PS Stage Embryo Shown in Figure 3
Embryo imaged in brightfield mode from the dorsal side. The primitive streak region can be seen extending horizontally.
(1.44 MB MOV)

Video S3 Movements of Labeled Epiblast and SE ECM during PS Formation Simultaneous imaging of H2B-GFP-labeled cells (top) and immunofluorescent fibronectin (bottom) in a single embryo (Figure 4) reveals concerted movements of epiblast and SE ECM during primitive streak formation. Note the striking similarity between convergence and extension movements in both components.
(5.57 MB MOV)

Video S4 Cellular Trajectories Relative to Immunofluorescent FN Cellular trajectories (Figure 4, right column) are overlaid on the immunofluorescent fibronectin (SE ECM) time-lapse image sequence. Note the close correspondence of cell tracks (colored lines) with the SE ECM movements.
(4.38 MB MOV)

Video S5 Click here for additional data file. High-Resolution Movie #1 of Labeled Epiblast Cells and Immunofluorescent FN
High-resolution time-lapse movie showing H2B-GFP-labeled cells (blue) and immunofluorescent fibronectin (green) in a relatively slow moving region of the epiblast corresponding to * in Figure 3.
(4.35 MB MOV)

Video S6 Click here for additional data file. High-Resolution Movie #2 of Labeled Epiblast Cells and Immunofluorescent FN
High-resolution time-lapse movie showing H2B-GFP-labeled cells (blue) and immunofluorescent fibronectin (green) in a relatively fast moving region of the epiblast corresponding to ** in Figure 3.
(3.79 MB MOV)

## Abbreviations

ACF: auto-correlation function
CA: cell-autonomous
ECM: extracellular matrix
FN: fibronectin
GFP: green fluorescent protein
HH: Hamburger-Hamilton
mAb: monoclonal antibody
PIV: particle image velocimetry
PS: primitive streak
SE: sub-epiblastic
VMP: virtual material particle(s)
